# Molecular docking analysis of CDK-1 inhibitors from Chrysophyllum cainito leaves

**DOI:** 10.6026/97320630017550

**Published:** 2021-05-31

**Authors:** Yesudass Antony Prabhu, Praveen Kumar Kumar, Shanmughavel Piramanayagam, Muthu Vijaya Sarathy, Samiappan Kavitha

**Affiliations:** 1 Department of Biochemistry, Rathnavel Subramaniam College of Arts and Science, Coimbatore - 641402, India; 2 Department of Bioinformatics, Bharathiar University, Coimbatore - 641046, India; 3 Department of Microbial Biotechnology, Bharathiar University, Coimbatore - 641046, India

**Keywords:** CDK-1, Oral squamous cell carcinoma, Chrysophyllum cainito, Schrodinger

## Abstract

It is of interest to document the molecular docking analysis of Cyclin-dependent kinase 1 (CDK-1) inhibitors from Chrysophyllum cainito leaves towards the treatment of tumors using the known structure of PDB ID: 5HQ0. Data shows that molecules such as 8- (Dimethylamino)-7-(3-(4-ethylphenoxy)-2d, ethyl 6-oxo-5-propylheptanoate, 2,3-dihydro-3, 5-dihydroxy-6-methyl-4h-pyran-4-one, 1,2,3-benzenetriol and 1,4-benzenediol 2,5-bis (1,1-dimethylethyl) identified in methanolic extract of C. cainito have binding features with CDK1 for further consideration.

## Background:

The sixth most common cancer is oral squamous cell carcinoma (OSCC), which affects 90% of people worldwide [1]. The mortality rate is high and the 5-year survival rate of OSCC cases remains low despite improvement in multidisciplinary treatment [2]. Dysregulation of CDK1 is frequently observed in many cancers, including OSCC patients. Because of this rationale, the relative 5-year survival rate of OSCC cases remains within 50-55% and lack disparity even after multidisciplinary treatment strategies including radiation therapy, surgery, and chemotherapy or combined therapy [[3]. Paclitaxel, an effective anti-cancer drug well known for the treatment of breast, neck, ovarian and lung cancer, is isolated from the tree bark of Taxus brevifolia [4].

Many medicinal plants with indigenous anti-cancer properties have been identified, but the underlying molecular mechanism is not well explored. In this way, the tropical tree Chrysophyllum cainito (C. cainito), is well recognized with anti-cancer effects using its stem extract, but the active agent responsible for exerting anticancer properties in the human liver cancer cell line is unresolved [5-6]. Schrodinger suite is used for molecular docking in this study. Therefore, it is of interest to find out the phytochemical profile of C. cainito leaf extract. And the anti-CDK1 inhibitory potential of bioactive compounds found in the methanolic extract was revealed through the molecular docking approach.

## Materials and methods: 

### Plant material:

The healthy fresh plants of C. cainito were collected from latitude 11.01° N, longitude: 76.95° E of Coimbatore District, Tamil Nadu, India. The plant species were identified and authenticated by the Botanical Survey of India (voucher number: BSI/SRC/5/23/2020/Tech/808), Tamilnadu Agricultural University campus, Coimbatore. The voucher specimen was provided with a scientific name and deposited for their future references.

### Simultaneous distillation and extraction from plant material:

C. cainito leaves were crushed to a fine powder after instant washing of leaves in tap water and distilled water, dried in the shade at room temperature for one week. Then the dried plant leaves were pulverized and stored in a jar until needed. The 100 grams of powdered C. cainito leaves were subjected to methanolic extractions (at 1:10 ratio) using the soxhlet apparatus at 65°C and proceeded for 12 hours over boiling percolation. The residual compounds of methanol extract were obtained by solvent evaporation by placing them in a hot air oven for two days at 50°C. The dark brown colour residues were then collected and used for further analysis.

### Phytochemical analysis using GC-MS:

The phytochemical analysis of a methanolic extract from C. cainito leaves was performed using GC-MS equipment (Thermo MS DSQ II, Thermo Fisher Scientific, USA). Before analysis, the samples were filtered using a 0.22 µm nylon syringe filter (Himedia, India). The instrumentation analysis employed through the following conditions such as DB 35-MS capillary non-polar column with dimensions 30 mm x 0.25 mm ID x 250 nm film thickness were used. Helium gas was used as a carrier, which is set at a flow rate of 1.0 mL/min. The injector temperature was operated at 250°C and the GC oven temperature was programmed from 60°C for 15 min, with a gradual increase of 12°C/min up to 280°C which is maintained and ending at 3 min. Finally, the mass spectra data were interpreted by comparing their analyzed retention indices of unknown components, with the known components of the NIST and Wiley spectral library databases [7]. Active site residues for the CDK-1 protein structure (5HQ0) were identified using the CASTp server. The residues are TYR 15, GLU 81, PHE 82, LEU 83, ASP 86, HIS 120, ARG 123, VAL 124, LEU 125, GLN 132, GLN 136, ASP 146 ARG 151, PHE 153, GLY 154, GLU 173, SER 178, ARG 180, TYR 181, SER 182, THR 183, PRO 184, ILE 187, TRP 228, PRO 229, GLU 230, VAL 231, LEU 234, ASP 271, PRO 272, ALA 273, ARG 275, ILE 276, SER 277, GLY 278 respectively.

### Molecular Docking:

A molecular docking study was performed using the Maestro module with Schrodinger docking suite to predict an interaction between specific ligand molecules and the binding sites of desired protein (5HQ0). [8]. In specific, the interaction between receptor CDK-1 (PDB ID: 5HQ0) and active molecules of C. cainito was anticipated using the Glide score [9]. There are two modes of docking calculations: XP (Extra Precision) and an SP (Standard Precision) method. Thus concerning the accuracy, the Glide module of XP visualizer connected with OPLS-2005 force field was used in this study to estimate the binding affinity. Table 1(see PDF) describes the molecular docking interactions of C. cainito onto the receptor 5HQ0 (Table 3 - see PDF).

### Preparation of Protein:

**Figure 1:**
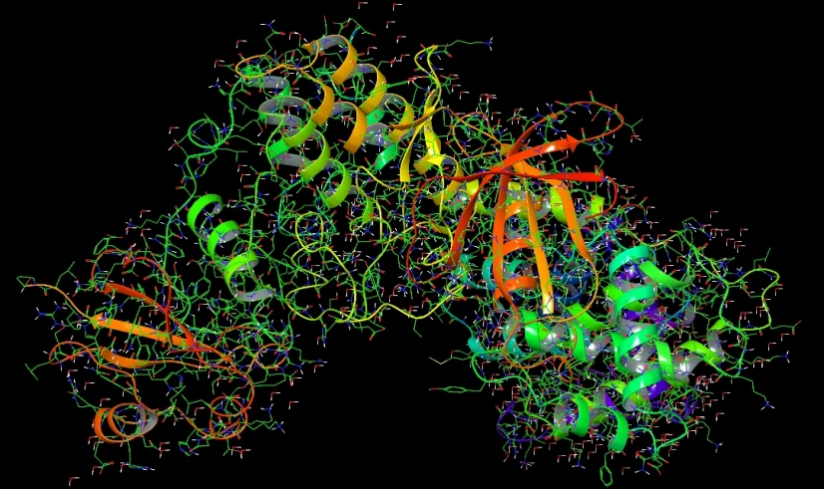
3D structure of the CDK-1 protein (PDB ID: 5HQ0). Source: RSCB Protein Data Bank, 2018. https://www.rcsb.org/structure/5HQ0

**Figure 2:**
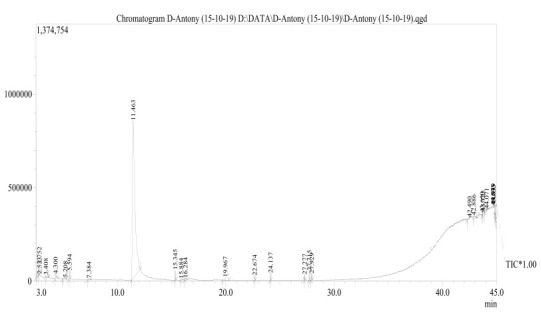
GC-MS chromatogram of methanolic extract of C. cainito leaves

The crystal structure of a CDK-1 domain was obtained from the Protein Data Bank (PDB) website (https://www.rcsb.org/), ensures with PDB ID: 5HQ0. The target protein was prepared using the protein preparation wizard tool (Schrodinger Suite, 2018). Briefly, for docking studies, the input protein molecule (CDK-1) were prepared with respective wizard applications such as deletion of unwanted chains and waters, fixing the orientation of hetero groups which are incorporated into the raw PDB structure. The generated 3D structure of CDK-1 protein was shown in (Figure 1). The 3D structures of input ligand molecules were retrieved from the PubChem database based on GCMS findings, and the structure was prepared using the LigPrep module [10] in Schrodinger Suite 2018. LigPrep tools contain options for default parameters such as tautomers, selective ionization states, stereo chemistries, tautomeric combinations, low energy structure and correct chiralities, ring conformations, the addition of hydrogen atoms, and versatile filters to create entirely customizable ligand libraries that are tailored for numerical analysis. Further, OPLS 2005 [11] was used to minimize and optimization of ligands. The phytochemicals discovered from a methanolic extract of C. cainito are shown in (Figure 2 and Table 2 - see PDF).

### Analysis of ADME/T property: 

QikProp module in Schrodinger software was used to assess ADME/T properties of those five compounds, which is shown in Table 1(see PDF). Because of the low ADME properties, several drug candidates fail in clinical trials. Thus QikProp creates valid identifiers for predicting significant physicochemical descriptors and pharmacokinetic relevant properties, of the ligand molecules (Table 1 - see PDF)[12]. The ADME/T properties of the ligand molecules determined through Lipinski's rule of five.

### Results and Discussion:

After successful methanolic extraction of C.cainito leaves in the soxhlet system, GC-MS analysis leads to the identification of many compounds through matching with the spectral library. Several compounds of varied chemical nature and structure eluted at different time intervals were detected in mass spectra with unique m/z ratios are shown in Figure 2. Thus the determined composition of methanolic extract corresponds to 100% of the entire GC-MS chromatogram. Further investigation under in-silico analysis elucidates the identification of structure and pharmacological property of potential molecules. Moreover, the present research focuses only on the identification of CDK-1 inhibitors rather than concerning the active form of CDK-1 protein necessitates the goal-oriented with drug discovery and drug development process.

The binding capacity of bioactive compounds from C. cainito leaves on CDK-1 protein associated with OSCC was investigated using Glide score. Glide score is an effective scoring function to estimate affinities of ligand-protein binding. Glide score ranking constitutes a docking algorithm in terms of the similarity penalty or through force fields scores (van der Waals, electrostatic) of ligand-binding interactions. However, the virtual screening of top-hit ligand-protein interactions entails precision docking, database enhancement and binding affinity to predict the best interaction score. And the total interaction energy of ligand-protein complex is an approximation of free energies of binding; therefore more negative values represent strong binders.

**Figure 3:**
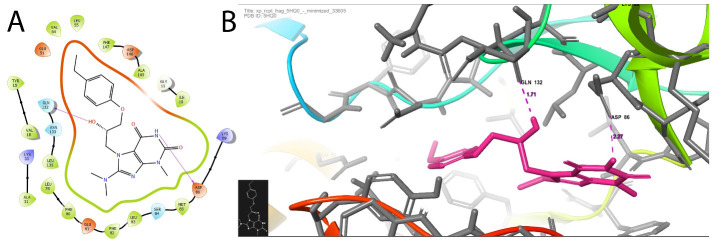
(A) displays the interaction of 8-(Dimethylamino)-7-(3-(4-ethylphenoxy)-2d over CDK-1 (PDB ID: 5HQ0) is shown. Hydrogen bonds specified as purple dash in individuals images. (B) The schematic representation of molecular interaction of an 8-(Dimethylamino)-7-(3-(4-ethylphenoxy)-2d over the CDK-1 protein (PDB ID: 5HQ0) is displayed.

Figure 3 emphasises the list of ligands from the C. cainito that are used in this in-silico study. Table 3(see PDF) displays the ligand interactions on CDK-1 protein (5HQ0) based on the factors such as Glide score, Dock Score, and H bonds. Notably, only five ligand molecules from C. cainito leaves extract have shown good interaction with 5HQ0 also shown in (Table 2 - see PDF). The docking results of phyto compounds and CDK-1 are described (Table 4 - see PDF). Of those ligands, 8-(Dimethylamino)-7-(3-(4-ethylphenoxy)-2d revealed the best docking score of -7.2 kcal/mol.

The bonding interactions of 8-(Dimethylamino)-7-(3-(4-ethylphenoxy)-2d and 5HQ0 were seen in (Figure 3A&B). It clearly suggests that the strong hydrophobic interactions are due to an oxygen atom of CDK-1 and the hydrogen atom of phyto compounds at its amino acid residues GLN132, ASP86 which better attained a bonding distance between 1.71 Å and 2.27 Å. Hence these ligand-5HQ0 interaction reveals good inhibitory activity against CDK-1 domain, becomes appropriate molecule in controlling OSCC progression.

Gscore of ensuing four ligands as follows: ethyl 6-oxo-5-propylheptanoate, 2,3-dihydro-3,5-dihydroxy-6-methyl-4h-pyran-4-one, 1,2,3-benzenetriol and 1,4-benzenediol 2,5-bis(1,1-dimethylethyl were -6.9, -6.53, -6.45 and -6.28. Oxygen bond of ethyl 6-oxo-5-propylheptanoate dichotomously found to be interacted on the complex of 5HQ0, remarkably at the residues of ASP 146 as well as an LEU 83 on hydrogen atom with the bond length of 1.97 Å and 2.06 Å. Following ligand 2,3-dihydro-3, 5-dihydroxy-6-methyl-4h-pyran-4-one hydrogen as well as oxygen bonds have found to possess the feasibility to interact simultaneously on the LEU 83 residue of the complex over oxygen and hydrogen bonds at the bond length of 2.12 Å 1.94 Å. Similarly, 1,2,3-benzenetriol and 1,4-benzenediol 2,5-bis(1,1-dimethylethyl have also found to interact with LEU 83 and GLN 132 residue with the bond length of 2.08 and 1.82.

## Conclusion:

We document the molecular binding features of 8-(Dimethylamino)-7-(3-(4-ethylphenoxy)-2d with the CDK-1 protein for further consideration.
